# Opportunities for precision livestock management in the face of climate change: a focus on extensive systems

**DOI:** 10.1093/af/vfab065

**Published:** 2021-10-20

**Authors:** Thomas Williams, Cara Wilson, Peter Wynn, Diogo Costa

**Affiliations:** 1 Institute for Future Farming Systems, Central Queensland University, Rockhampton, QLD, Australia; 2 School of Animal and Veterinary Sciences, Faculty of Science, Charles Sturt University, Wagga Wagga, NSW, Australia; 3 EH Graham Centre for Agricultural Innovation, Charles Sturt University, Wagga Wagga, NSW, Australia

**Keywords:** carbon abatement, extensive livestock systems, greenhouse gases, precision technologies

**Implications**

- Precision livestock management (PLM) technologies will allow producers to learn more about the limits to production efficiency on their enterprise.
- From this information, producers will be able to make more assertive decisions to improve enterprise efficiencies and reduce greenhouse gas emissions.
- PLM technologies will allow producers to access new markets and incentive programs which will increase enterprise revenue.
- Leveraging PLM technologies will increase the environmental sustainability of enterprises, ensuring producers retain social license to operate.
- Future software will need to be capable of integrating the multiple data streams being produced to maintain on-property financial and environmental resilience.

## Introduction 

Climate change and carbon neutrality have become key topics in extensive livestock industries in recent years as mankind attempts to meet its commitments to restricting global warming (United Nations, 2015). Initial impressions are that extensive livestock systems involving low cattle stocking rates over large rangeland areas are inefficient and produce more greenhouse gases (**GHG**) per harvested animal compared with intensive systems. Extensive livestock enterprises are often located in dry geographical areas with increasingly inconsistent rainfall, making them vulnerable to the impacts of climate change (Huang et al., 2017). Changing and more variable climates require producers to adapt their management practices to remain sustainable and increase enterprise resilience (Rust, 2019). In these environments, understanding the relationship between nutrient availability and animal performance is vital.

Key constraints to mitigating the effects of climate change in extensive livestock enterprises are the sheer scale of operations, their low input management strategies, and the logistical inability to collect production and environmental data on a regular basis. However, the advent of the precision technology revolution has provided a wide range of opportunities to capture high-value data in these extensive livestock enterprises. This is being realized in intensive livestock systems (González et al., 2018) and cropping enterprises (Shafi et al., 2019; Oliveira et al., 2020) but is yet to reach its full potential in the extensive livestock sector.

Advances in the genetic potential of beef cattle, and the incorporation of *Bos indicus* breeds, have extended the reach of beef enterprises into more marginal agricultural regions. The question now remains how we can utilize these cattle to generate profits for producers from these highly variable rangelands through precision livestock management (**PLM**) while at the same time conserving this fragile ecosystem. Subtropical and tropical grasslands account for a substantial portion of the world’s available arable land. Humankind must continue to improve sustainable food production systems to feed the world’s projected 9.8 billion people by 2050. Our objective is to identify the major advances in PLM technologies that allow mankind to achieve this outcome.

### The scope of PLM

PLM is not a new concept. It has allowed the beef producer to progress from being paid dollars per head, to dollars per kilogram carcass weight, and from locating cattle via the sound from a cowbell to the use of Global Positioning System (GPS). Although PLM has developed over time and greatly increased productivity in intensive livestock systems, the next generation of PLM technologies, such as on-animal sensors (i.e., “smart-tags”), off-animal sensors (i.e., walk-over-weigh), and remote sensors (i.e., satellite imagery), will provide opportunities for extensive livestock industries to significantly increase the granularity of data and allow management decisions to be based on a greater diversity of more informative descriptors of production efficiency. Reductions in labor requirements also cannot be discounted. PLM technologies provide livestock enterprises with high-resolution and high-frequency data streams which are currently difficult or unrealistic to obtain in extensive systems. PLM technologies also exist for the physical management of cattle in extensive systems. Commercially available management options include automated drafting, virtual fencing, and drone mustering. These technologies are also likely to be beneficial through labor reductions. However, in this article, we focus on technologies that increase the granularity of production system information to inform management decisions.

Berckmans (2017) suggested that PLM technologies will deliver increased efficiency and sustainability in livestock production while also improving welfare outcomes and enabling traceability of product along the supply chain. Despite slow progress over the last decade, demand-led innovation has seen the formation of numerous AgriTech businesses that supply PLM products into the extensive livestock marketplace, suggesting a potential period of industry emergence and maturation.

A developing market exists for PLM technologies in mitigating the impacts of climate change in extensive livestock enterprises. Government organizations and industry bodies are increasingly developing policy that moves toward carbon neutrality (Alves et al., 2017; Slade, 2018; Red Meat Advisory Council, 2020). These policies either incentivize carbon reduction or penalize GHG emissions. PLM technologies can lead the way under both incentive and penalty scenarios through improving production efficiencies, automating access to carbon abatement schemes, or increasing market access through validating climate positive livestock products.

## Sustainable Production through Resilient Livestock Enterprises

PLM technologies allow for objective and more frequent observation of traditional performance measures. Using PLM technologies, there is potential for producers to know the location of their livestock (Bailey et al., 2018) or be alerted during dog predation events (Manning et al., 2014) using GPS. Producers can measure how their livestock are performing using walk-over-weigh or partial-weigh technologies in real time (Menzies et al., 2018; Cantor et al., 2020), and any disruptions to performance can be investigated using accelerometers to assess physical activity which may be indicative of disease (Tobin et al., 2020), birth events (Chang et al., 2020; Fogarty et al., 2020), or even to monitor rumination behavior (Wolfger et al., 2015) and predict feed intake (Greenwood et al., 2017). Supplementary data streams that provide environmental information are also available for integration (Fogarty et al., 2021). For example, multispectral imagery can estimate the amount of available forage at varying spatial resolutions (Handcock et al., 2016), and weather station mesh networks can provide information to predict pasture growth at the sub-paddock level and alert producers to risk periods for heat stress. Past the farm gate, on-animal sensors can provide information on the impact of extreme heat or cold events during transport and lairage (Rashamol et al., 2019). These technologies are a reality now, and commercial devices are becoming available for incorporation into extensive livestock enterprises.

The greatest value from PLM technologies will be realized when combinations of data streams across a property and supply chain are leveraged to inform decision-making. Research is yet to generate models that could be incorporated into commercial platforms, but increasingly, authors are noting their potential (Tedeschi et al., 2021). Through analysis and predictive modeling, integrated PLM data streams could provide producers with an information-dense online interface that offers real-time data pertinent to maintaining a high-efficiency enterprise, thus reducing GHG emissions.

PLM data streams with interpretive modeling could monitor, forecast, and validate livestock productivity and feed base availability at a sub-paddock level. These data would inform day-to-day management, accurately estimating viable stocking rates and providing rapid alerts where interventions such as paddock movements or supplementation are required to maintain sustainable levels of productivity. Forecasted production data could also alert producers when livestock are approaching exit weights, ensuring producers are not penalized for missing specification or maintaining livestock unnecessarily. The removal of individual animals not achieving production specifications from extensive beef production enterprises will assist with enterprise profitability. Using the phenotypic data captured through whole-of-system and supply chain monitoring, producers could select for highly efficient and resilient livestock within their enterprise. These data could also be incorporated into genetic evaluation platforms to progress the genetic potential of the national herd. Traits that are difficult to capture in extensive enterprises such as reproductive performance could become easily accessible, and new traits, specific to PLM technologies, such as grazing distribution preference would become available (Bailey et al., 2006). In effect, the incorporation of PLM technologies into extensive enterprises will provide producers with the opportunity to assess whole-of-system performance and use the captured data for benchmarking, identifying GHG emission inefficiencies, and increasing enterprise resilience.

## Accessing New Revenue Streams and Retaining Social License to Operate

### Carbon abatement programs

Livestock systems are responsible for approximately 15% of the global GHG emission (Gerssen-Gondelach et al., 2017). Abatement programs provide opportunities for producers to increase income through management and measurement of carbon emissions and sequestration within their enterprise. One example of this is offered through the Emissions Reduction Fund (**ERF**) in Australia (Clean Energy Regulator). The ERF’s beef cattle herd management project incentivizes high-efficiency livestock production through regular monitoring of livestock growth rates and comparison to a standard growth rate (Department of the Environment and Energy, 2017). The method emphasizes the production of faster-growing livestock, which in turn will produce less GHG emissions during their time in extensive production systems (Mayberry et al., 2019). The current method for capturing project production data requires livestock to be weighed up to four times per year. Although most weighing events will typically coincide with other management interventions, there is scope to increase the frequency and ease of data capture with validation using PLM technologies such as walk-over-weigh or partial-weigh systems. Incorporating integrated PLM technologies into beef production systems can support decision-making to reduce climate impact on-farm, improve efficiency, and subsequently achieve levels of production that qualify for ERF incentivization. PLM technologies often have preexisting database infrastructure that would allow fast and secure transfer of performance data to carbon abatement schemes, giving multiple producers rapid access as part of their service.

Meat and Livestock Australia predicts that approximately US$60 million will be invested by 2030 on initiatives to capture revenue from carbon credits alone based on their key performance indicators, with a benefit:cost ratio of 13:1 as the potential investment return for each dollar (Red Meat Advisory Council, 2020). Key constraints to accessing these incentive programs are the difficulty for producers to register and subsequent regulatory on-costs. In some incentive schemes, carbon credits can be aggregated across multiple enterprises. Aggregation can be undertaken by a third party more capable of the regulatory environment, reducing the access barriers for producers.

**Figure 1.**
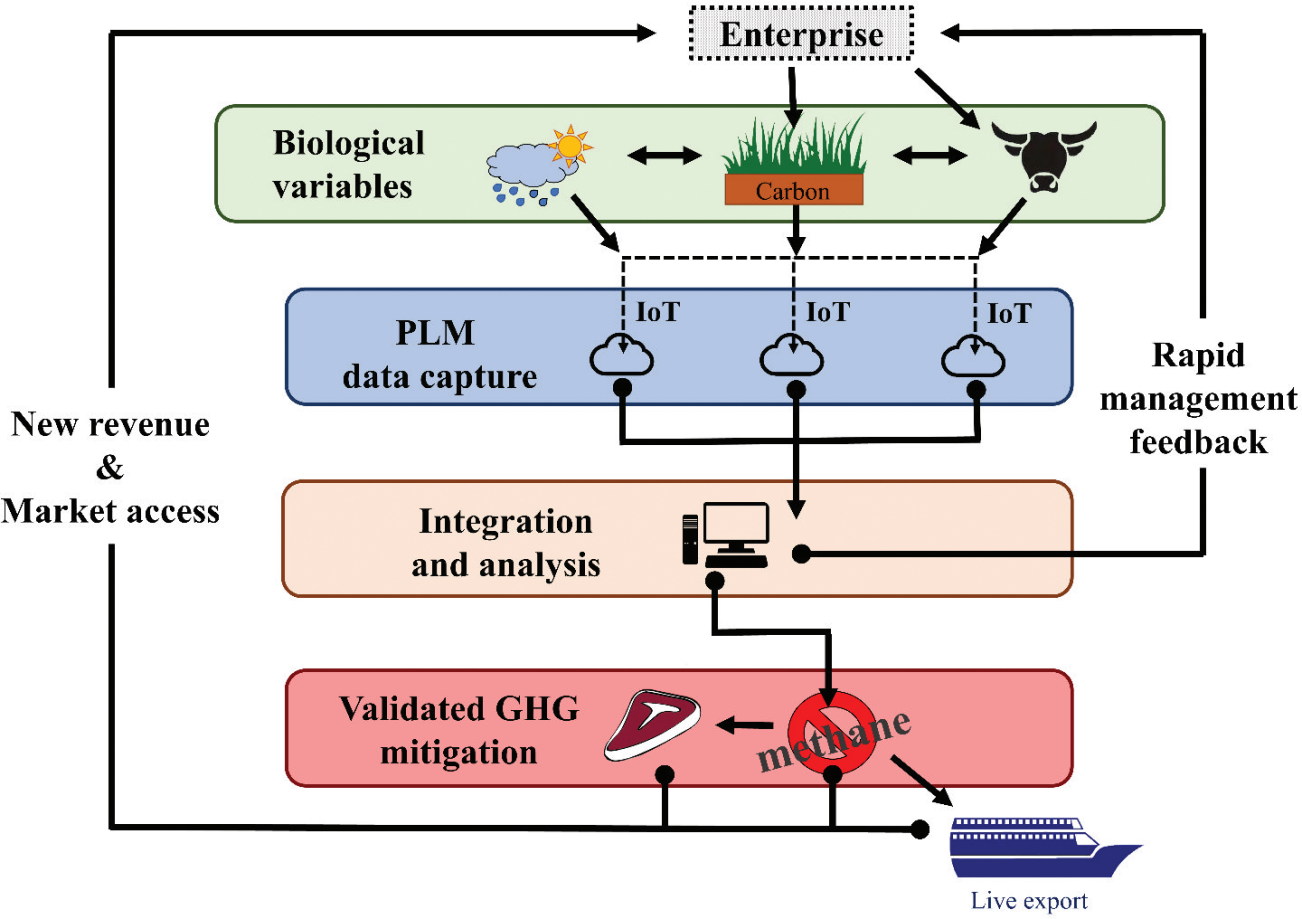
Summarized approach of data from precision technologies’ integration in carbon abatements program. Abbreviation: IoT, Internet of things.

In contrast to substituting traditional measurement techniques, opportunities exist for PLM technologies to become the key measurement tool for carbon abatement schemes. As previously described, high-resolution PLM data could be used to inform key measures of enterprise efficiency and employed to estimate subsequent carbon emissions where other types of measurement are impractical. Key metrics such as pasture intake (Greenwood et al., 2017), animal activity (Chang et al., 2020), liveweight, mortality, and reproductive performance (Menzies et al., 2018) could be used to calculate an individual animal’s carbon emissions index, with benchmarking and carbon abatement amounts being calculated daily. A simplified diagram (Figure 1) summarizes the concept of adoption of PLM in conjunction with GHG abatement programs.

### Reducing economic barriers to trade

Livestock industries must address the issue of GHG emissions or risk losing market access and social license to operate (Ramirez, 2018; Red Meat Advisory Council, 2020). Access to new markets through branded products such as paddock-to-plate, grass-fed, organic, and high-welfare certification has increased product value for some producers. Climate-smart animal products have begun to enter the market and indications suggest that demand will increase for ethically sourced products (Le, 2018). The North Australian Pastoral Company (NAPCo) has launched a carbon neutral product, “Five-Founders” (North Australian Pastoral Company, 2021), while the Brazilian Agricultural Research Corporation (EMBRAPA) has promoted the system-wide concept of “Brazilian Carbon Neutral” beef (Alves et al., 2017). In contrast, in Canada, the government announced a raise in tax for high emission products, such as beef, as a stimulus for consumers to shift their consumption to lower emission alternatives (Slade, 2018). Consumers demand high transparency along the supply chain, and PLM technology could act to validate certification claims and provide consumers with access to end-product emission estimates from across the supply chain, with particular focus on extensive livestock systems (Alfian et al., 2017).

## Research and Development Focus Moving Forward

Despite increasing activity in the sector, the AgriTech industry has many limitations to overcome before becoming commonplace in extensive livestock enterprises. The two key constraints—hardware limitations (including connectivity) and tangible value to producers—continue to limit uptake and adoption of PLM technologies. Research in the sector has identified multiple opportunities to improve the value proposition of AgriTech in extensive livestock industries (Bailey et al., 2018), but the incorporation of findings into commercially available technologies has been limited. Often their incorporation into the extensive industry has been considered at a higher, industry-wide level with little consideration for the end user who must ultimately make the decision to invest in products on offer. These views have resulted in poor adoption and uptake by producers. The PLM technologies showing greatest promise will be those with tangible, real-time value for producers, while providing climate positive outcomes. This value could be added through devices having several applications, for example preventing stock theft, monitoring grazing patterns of livestock, and detecting adverse health or welfare events. Devices need to be quick and simple to apply to fit in with normal husbandry procedures and they must be accessible for everyone, not just those with good connectivity. Adoption among producers is improved when they have access to accurate information. Considering social factors is also important. Therefore, extension activities should be targeted at well-respected producers in specific areas (Liu et al., 2018). Climate change policy will continue to develop, and extensive livestock operations will be impacted. Dependent on strategies for carbon abatement, PLM technologies will inform graziers on opportunities to increase system efficiency or allow access to additional revenue streams. To do this, research and development within the scientific and commercial sectors need to focus on opportunities for climate impact validation. Moving forward, research should look to integrate the multiple data streams being produced on-property to allow producers to make the right decisions to minimize climate impact on-farm. Whole-of-system integration will increase opportunities for carbon abatement. To realize these opportunities, development should be undertaken collaboratively between scientific and commercial sectors, ensuring fit-for-purpose hardware, models, and software attractive to the end user.

## About the Authors

**Dr Thomas Williams** completed his undergraduate and postgraduate studies at Charles Sturt University before working as a Postdoc within the large ruminant health team. His research has focused on established and developing industries, assessing the interactions between parasites, wildlife hosts, farming practices, and livestock disease. More recently, his work has investigated novel therapeutics and point-of-decision diagnostic tools for improved cattle health and production outcomes in extensive and intensive systems. In 2020, he joined the Precision Livestock Management group at CQUniversity where he now works developing remote monitoring technologies for the Northern Beef Industry. His interests include improving efficiency and welfare in extensive livestock systems using emerging precision technologies.

**Cara Wilson** is a Research Officer in the Precision Livestock Management team at Central Queensland University in Rockhampton. Her research focuses on the utilization of AgriTech Technologies in livestock traceability systems and using these technologies to detect livestock health and welfare issues throughout the value chain. Integrating data from pre-farm gate to abattoir and beyond has the potential to improve our biosecurity systems and market access. She also works as a STEM Project Officer where she develops and implements programs centered around getting children excited about STEM and thinking about careers in STEM. She has completed a BEqSci, BSc (HonsI), and PhD at Charles Sturt University in Wagga Wagga. Her passions are conducting research that provides practical and sustainable outcomes to industry and sharing the results with the wider community.

**Peter Wynn** is an Adjunct Professor who holds a PhD from the University of Sydney and has developed a career in teaching and investigating limitations to the productivity of grazing ruminants and pigs at Sydney and Charles Sturt Universities after periods of research on wool and growth physiology with Commonwealth Scientific and Industrial Research Organisation and on the physiology of stress at the National Institutes of Health Bethesda Maryland. In recent years, he has led an Australian Centre for International Agricultural Research program to support extension services for small-holder dairy farmers in Pakistan.

**Diogo Costa** is a Senior Research Officer at Central Queensland University. His passion for agriculture and livestock led him to study agronomy at the University of Sao Paulo (USP). His involvement with research started there and continued to develop over the years with a Masters at USP and a PhD at the University of Queensland. Over the past 20 yr, he has developed extensive experience in a research career focusing on cattle production in grazing and feedlot systems. He is currently working on a project looking into the use of cutting-edge technology for sensing calving and calf loss. Gathering data to help producers address perinatal calf mortality remains a significant challenge in extensive grazing landscapes, and his main goal is to evaluate and enhance tools and systems to provide this critical missing information, working directly with producers to identify their needs and deliver practical solutions to the livestock industries.
